# Interpretation and the art of care— Gadamer’s hermeneutics and physician wellbeing

**DOI:** 10.1186/s13010-026-00215-4

**Published:** 2026-06-02

**Authors:** Peter Shannon

**Affiliations:** 1 American Institute for Music and Healing, Martin, USA; 2 Department of Bioethics and Medical Humanities, Mercer University, Macon, USA

**Keywords:** Health humanities, Medical humanities, Hans-Georg Gadamer, Philosophical hermeneutics, *Truth and Method*, *The enigma of health*, Burnout, Resilience, Moral injury, Doctor-patient relationship

## Abstract

Physician burnout has increasingly been associated not only with workload and administrative burden, but also with a perceived erosion of meaning and professional identity within contemporary medical practice. This article explores the potential of Hans-Georg Gadamer’s philosophical hermeneutics as a resource for reorienting physicians toward a more sustainable practice of medicine. The present argument does not propose hermeneutic awareness as a singular solution to burnout, but rather as a conceptual lens through which one important dimension of burnout — the experience of diminished meaning — may be understood. Key elements of Gadamer’s philosophy as outlined in *Truth and Method* Gadamer (Truth and method (2., rev. ed). Continuum 2003) and *The Enigma of Health* Gadamer (The enigma of health: The art of healing in a scientific age 1996) are presented here as a conceptual framework, inviting the doctor to reconsider the doctor-patient dialogue from a *new* horizon—one shaped by a curiosity to know more about the person sitting across from them. Existing scholarship in the phenomenology of medicine has emphasised the interpretive nature of clinical practice; the present article extends this discussion by considering how hermeneutic awareness may also support physician wellbeing through renewed attentiveness to relational and interpretive dimensions of care. Reflective exercises are offered as illustrative applications of Gadamerian concepts within contemporary clinical contexts. While structural causes of burnout require systemic solutions, a hermeneutic perspective highlights how meaning, dialogue, and interpretive attentiveness may remain accessible within everyday clinical encounters. In this way, Gadamer’s philosophy contributes to ongoing efforts within the health humanities to re-articulate medicine as both a scientific and interpretive practice.

## Background

The culture of modern healthcare has produced record levels of burnout and moral injury, fuelled by rising workloads, bureaucratic demands, and structures that, however well meant, undermine physicians’ capacity to fulfil their central vocation of patient care. Moral injury refers to the distress experienced when clinicians feel unable to act in accordance with their professional values due to systemic constraints [18]. While physician burnout rates in the United States have declined since the peak of the COVID-19 pandemic, they remained, as of 2024 ‘stubbornly high compared with those of other American workers’ [16]. In Ireland in 2021 the Irish Medical Organisation (IMO) published that a staggering ‘90% of doctors reported having experienced some form of depression, anxiety, stress, emotional stress or other mental health condition related to or made worse by work’ [15].

When clinicians are depleted, their ability to listen, understand, and care for patients diminishes in turn, revealing a ‘significant association between burnout and patient safety’ [13]. Higher levels of burnout have been associated with reductions in physicians’ professional work effort over time, suggesting that emotional exhaustion may influence decisions to decrease clinical workload [26], exacerbating the already existing problem of reduced resources within the healthcare system. Emotional burnout has financial implications, with a 2022 report estimating that the cost of poor mental health and wellbeing to the NHS might amount to £12.1 billion per year [24]. Research shows that *when* healthcare professionals’ wellbeing is cared for, patient outcomes improve [25].

Burnout has been widely studied in contemporary healthcare and is generally understood as a syndrome involving emotional exhaustion, depersonalization, and a diminished sense of professional accomplishment [20]. Research has consistently linked burnout not only to workload and administrative pressures but also to a loss of meaning and connection within clinical work itself. Crucially, studies led by Tait Shanafelt and colleagues have shown that physicians who report higher levels of professional fulfilment and meaning in their work experience significantly lower levels of burnout, even when controlling for workload and other structural factors [26]. These findings suggest that while systemic pressures play a critical role in physician wellbeing, the experience of meaning and connection within the patient encounter remains a significant protective factor. This relational dimension is not merely an ethical or philosophical ideal but has measurable biological significance. As Sue Carter has shown, compassion and social connection are associated with improved health outcomes and even survival value, suggesting that the capacity to care for others is itself a fundamental component of human wellbeing ([3], p. 173).

Facing the reality that systemic changes are not within the individual control of the physician, physicians are tasked with finding ways to build resilience and protect one’s mental and physical health. Gadamer’s philosophical hermeneutics invites us to reimagine the clinical encounter, shifting our focus from technique to understanding, from procedure to participation, and from method to meaning. This article argues that doing so may offer a new and valuable reframing of the doctor–patient relationship, one that not only advances the health humanities but also deepens understanding between physician and patient, transforming the clinical encounter into a space of trust, empathy, and shared humanity that enriches both.

### Situating Gadamer within the Phenomenology of Medicine

The application of phenomenology and hermeneutics to medicine is not new. Scholars such as Fredrik Svenaeus have drawn on Hans-Georg Gadamer to interpret the doctor–patient relationship as a fundamentally hermeneutic encounter, arguing that health and illness must be understood through lived experience as well as biological explanation:

Medicine is an interpretive meeting, which takes place between two persons (the doctor or some other clinical professional and the patient) with the aim of healing the one who is ill through developing an understanding of her human predicament. Clinical medicine—and this is the sense in which the term ‘medicine’ will be used in this book unless otherwise specified—is thus first and foremost a practice and not a science’ [29].

This positions medicine not as the application of scientific knowledge, but as an interpretive practice grounded in human encounter. Similarly, Edmund Pellegrino approached clinical practice as a moral and phenomenological relationship grounded in trust and interpretation [22].

Kay Toombs has described how the patient’s experience of illness can feel like an alien bodily sensation, producing a sense of loss of control, while the physician approaches illness from the more distanced perspective of biological processes, pathogens, and physiological mechanisms. Writing from a phenomenological perspective informed by her own experience as a patient with multiple sclerosis, Toombs observes:

My interest in exploring the nature of the patient’s and the physician’s understanding of illness has grown out of my own experience as a multiple sclerosis patient. In discussing my illness with physicians, it has often seemed to me that we have been somehow talking at cross purposes, discussing different things, never quite reaching one another. This inability to communicate does not, for the most part, result from inattentiveness or insensitivity but from a fundamental disagreement about the nature of illness. Rather than representing a shared reality between us, illness represents two quite distinct realities - the meaning of one being significantly and distinctively different from the meaning of the other [30].

This fundamental difference can understandably create communication challenges and a sense of alienation for the patient. Toombs’ account comes closest to exposing the experiential divergence between patient and physician.

Gadamerian concepts have informed scholarship in medical education, where interpretive awareness and reflective dialogue are central to professional formation and ethical clinical practice [19]. Gadamerian approaches have also informed educational initiatives in the health humanities beyond the biomedical model, including programmes developed through the American Institute for Music and Healing (AIMH), where music — especially listening — functions as a modality for engaging core hermeneutic concepts of dialogue, openness, and interpretive awareness [27]. Gadamer’s hermeneutical outlook has also been applied to clinical interpretation, including the ‘translation’ of patient experience into medically meaningful symptoms. The ‘fusion of horizons’ has been used to conceptualise the consultation as an interpretive encounter in which meaning emerges dialogically between patient and physician [5].

However, while illuminating the interpretive and relational dimensions of the doctor–patient encounter, these approaches largely remain focused on the patient’s experience or the epistemology of care, leaving underexplored how these same dynamics shape the physician’s own experience of meaning, purpose, and wellbeing.

Building on this work, the present article explores Gadamer’s hermeneutics specifically in relation to physician wellbeing, arguing that the interpretive and dialogical dimensions of clinical care may not only deepen understanding of the patient but also help restore meaning and connection for physicians themselves. Crucially, this is not a separate concern: a growing body of evidence suggests that physician wellbeing is itself closely linked to the quality of patient care, such that restoring meaning for the clinician is inseparable from improving outcomes for the patient [31].

### Hermeneutics of healing: reframing the doctor–patient relationship through Gadamer’s Truth and Method

Hans-Georg Gadamer’s monolith *Wahrheit und Methode* (Truth and Method), published in 1960, was first translated into English in 1975 [12].However, it had to wait until 1989 to receive what is now considered the standard translation [11]. There is no doubt that engaging with *Truth and Method* requires sustained effort— the conceptual density of Gadamer’s arguments and the intricacy of his language present considerable challenges even to the native reader. Part of this difficulty lies in the highly idiomatic precision and stylistic subtlety of Gadamer’s German (he often plays with words which makes direct translation impossible) which resists seamless translation. Although the standard English edition is exemplary, the text nevertheless demands patience and, ideally, engagement with Gadamer’s other writings and essays for fuller comprehension.

Ironically, this very process of seeking understanding through multiple perspectives mirrors one of Gadamer’s central philosophical insights—his notion of *Horizontverschmelzung* which has been translated as ‘fusion of horizons’ (translated literally, *Horizontverschmelzung* is a ‘Melting of Horizons’), a concept that will be explored in greater detail later in this paper. Gadamer’s book *The Enigma of Health* (1996) is a compilation of lectures and essays on health, illness and healing [9]. It is essential reading for two reasons: while it doesn’t have any of the detail as to *why* Gadamer believes what he does (that is all painstakingly laid out in Truth and Method) it does embody all of Gadamer’s central philosophical principles. Also, (unlike *Truth and Method*) it speaks directly to the healthcare professional, illuminating the interpretive and relational dimensions of clinical practice and the doctor-patient relationship.

Gadamer’s title *Truth and Method* gets to the heart of what his hermeneutical outlook is—while acknowledging the extraordinary achievements made possible by ‘method’ in the natural sciences, he insists that there exists another, equally vital mode of knowing within the human sciences. This form of knowledge—rooted in interpretation, experience, and dialogue—cannot be measured in the same way the natural sciences are measured. This form of knowledge is one that emerges through our engagement with art, history, and human encounter. In fact, Gadamer challenges the notion that methodological procedures can definitively establish truth, contending that science constructs the very laws and criteria by which it validates its own claims. He gives the example of the school experiment where two items (a feather and a piece of lead) fall to the ground at the same speed, but only when placed in the unnatural environment of a vacuum.

The 2020 Harvard study that reported a dose-dependent association between prenatal acetaminophen exposure and increased risk of neurodevelopmental disorders, including ADHD and autism, and the ensuing debate in 2025, exposes just how timely Gadamer’s critique of modern science remains [23]. What we witnessed was not simply a disagreement over data, but a deeper tension between competing epistemological assumptions: one oriented toward isolating discrete causal factors, and another increasingly systemic, recognizing that health emerges from complex interactions between biology, environment, and lived context. Both sides claim the mantle of science, yet they operate within self-reinforcing frameworks that help determine what counts as valid evidence. In his chapter *‘*Treatment and Dialogue’ in *The Enigma of Health*, Gadamer warned that modern medicine’s reliance on method and measurement risks obscuring the interpretive and holistic character of health—that illness cannot be fully understood through isolated causal chains alone, but requires attention to the coherence of the living whole:

In the meantime, however, modern science has come to regard the results of such measuring procedures as the real facts which it must seek to order and collect. But the data provided in this way only reflect conventionally established criteria brought to the phenomena from without. They are always our own criteria which we impose on the thing we wish to measure ([9], p. 132).

The Tylenol debate thus exposes a methodological blind spot in modern medicine: an assumption that truth lies primarily in the quantifiable. There is more to this than meets the eye—it is more than a public health dispute; it reflects an underlying tension in scientific understanding, where the authority of method risks eclipsing the role of interpretation. This example illustrates how interpretation operates not only in clinical encounters but also in scientific discourse, reminding us that medical knowledge itself develops within historically situated horizons of understanding.

### Gadamer, and the doctor-patient relationship as a pathway to self-care

Gadamer has particular significance for the health humanities because his ontological outlook informs the idea that caring for a patient is not just about applying a method or technique, but engaging with their patients’ lived experience and engaging in dialogue that reveals their perspectives and priorities, thereby guiding a more responsive and individualised care. In the clinical setting, this means that beyond the measurable facts of diagnosis and treatment lies another, *equally essential form of truth*: the mutual understanding that arises between doctor and patient. Research in the health sciences increasingly confirms what Gadamer intuited: good communication and genuine understanding are not ancillary to healing, but *constitutive of it*. Gadamer’s approach is deeply embedded in phenomenology, in his conviction that the ‘truth’ in medicine lies in dialogue with the patient. This is more than ‘just talking’—it is real engagement in every sense of the word, and has parallels in recent research:

Although prior research indicates that features of clinician-patient communication can predict health outcomes weeks and months after the consultation, the mechanisms accounting for these findings are poorly understood. While talk itself can be therapeutic (e.g., lessening the patient’s anxiety, providing comfort), more often clinician-patient communication influences health outcomes via a more indirect route. Proximal outcomes of the interaction include patient understanding, trust, and clinician-patient agreement. These affect intermediate outcomes (e.g., increased adherence, better self-care skills) which, in turn, affect health and well-being [28].

The findings show that communication benefits patients, even when the mechanisms remain partly hidden or have ‘a more indirect route’. From a Gadamerian perspective, this is hardly surprising: not everything meaningful in human interaction can be measured. This article wishes to take a step further, by positing that through Gadamer’s hermeneutical outlook, the patient encounter can become more authentic and mutually enriching, sustaining the physician’s sense of purpose, and renewing the physician’s connection to their work. The following sections introduce key aspects of Gadamer’s philosophy and explore how they can inform and enrich healthcare practice.

### Historically-effected consciousness (*Wirkungsgeschichtliches Bewusstsein*)

One of the defining concepts in Gadamer’s philosophical outlook is the concept of ‘Wirkungsgeschichtliches Bewusstsein’, which can be translated as ‘historically effected consciousness’, or ‘effective historical consciousness’. Gadamer argues that all of our understanding is shaped by our place in history. Our understanding of all things is limited by the fact that we do not have access to the past or the future. We may *think* that we can understand an event in our history by placing ourselves within that mindset, arguing that history books and first-hand accounts from that time equip us to form an accurate assessment of an event in history, or the mindset of someone from that time. While Gadamer doesn’t say that understanding history is impossible—he says it is always interpretive, because we inevitably bring our own historically-situated perspective into the encounter.

In medicine, research is often described as ‘cutting-edge’, or as ‘leading technology’. Such language implies linear progress, yet what is considered advanced at one historical moment may later be reassessed in light of new knowledge. But this was true of every generation in the past. The case of thalidomide illustrates the stakes. Widely prescribed in Europe in the late 1950s, it caused devastating birth defects before its risks were fully recognised. In the United States, its approval was halted largely due to the insistence of FDA reviewer Frances Kelsey, whose caution saved countless lives.

Additionally, history repeatedly shows that transformative ideas in medicine often struggle to gain acceptance within established institutions. Early neonatal incubators—now standard in neonatal care—were initially dismissed by many hospitals. With little institutional support, physician–showman Martin Couney resorted to a striking solution: he set up fully functioning incubator wards at Coney Island amusement parks. Visitors paid to see fragile premature infants in glass incubators while nurses provided continuous care. What appeared as spectacle to the public was, in fact, a lifesaving medical intervention that preserved thousands of premature infants who otherwise might not have survived. More recently, immunologist James Allison faced repeated rejection of his research on immune checkpoint blockade from conventional cancer research funders. Undeterred, he persisted, ultimately revolutionizing cancer therapy and earning a Nobel Prize. These examples illustrate a recurring theme: medical progress often relies not only on institutional endorsement but on the persistence of individuals willing to challenge prevailing assumptions when evidence and insight point in a different direction.

For the medical professional, and in particular the biomedical model, which prides itself on progress and finding definitive answers, such a vantage point may lead to a feeling of powerlessness or inability. A depressing outlook? Not so. In Gadamerian terms, accepting the finitude of our understanding is not defeat but the beginning of genuine openness—an awareness that restores dialogue, curiosity, and care to their rightful place in medicine: ‘Health is not a condition that one introspectively scrutinizes, but rather a state of being involved, of being in the world’ [9].

The physician who adopts a *historically effected consciousness* acknowledges their place in history—in a continuum of caregivers who have always sought to heal, using the tools and knowledge available to them at that time. Rather than feeling constrained by the current limits of medical science, they recognise that no generation will ever possess all the answers:

The illumination of this situation—reflection on effective history—can never be completely achieved; yet the fact that it cannot be completed is due not to a deficiency in reflection but to the essence of the historical being that we are. *To be historically means that knowledge of oneself can never be complete (* [11], p. *302).*

By accepting that medicine’s methods—however rigorous—can also fail or fall short, the physician opens space for another dimension of care: *the art of medicine*. Here, meaning arises not from technical mastery but from relationship, from the willingness to engage with the patient as a partner in dialogue. In allowing that dialogue to ‘speak’ to them, physicians may come to understand not only more about the illness, but also about the patient—and, ultimately, about themselves. This reflexive dimension of understanding is central to Gadamer’s view that interpretation always involves the interpreter as well as the object of interpretation [11]. For the physician’s own self-care, this awareness allows doctors to separate self-worth from perfectionism, to view uncertainty as intrinsic to medicine rather than as failure. The doctor’s responsibility is not to have all the answers, but to bring wisdom, compassion, and attentiveness to the situation at hand. It allows for a humble confidence: *I am acting with integrity and understanding in this moment with the information and skills I have—and that is enough.*

The following sets of reflective practices are not intended as prescriptive psychological interventions, but as practical illustrations of how Gadamer’s philosophical insights may be translated into everyday forms of interpretive attentiveness. They are reflective exercises designed to cultivate awareness of historically effected consciousness, dialogue, and practical wisdom within the lived experience of clinical work.

### Reflective practice

- Reflect on the difference between personal identity and professional performance. At different intervals throughout the day spend a mindful moment reflecting with the words: *‘Even amid uncertainty*,* I am acting with integrity and care’.*
- Who in your family’s history has fostered/encouraged your qualities of healing in you? What are the characteristics of these qualities?
- What traditions, teachers, and assumptions shape how I understand health, illness, and success? What are the positive aspects of these and how can I mindfully integrate them into my practice?

### Dialogue and the ‘Fusion of Horizons’ (*Horizontverschmeltzung*)

For Gadamer, language is not merely a tool for communication, but the very medium through which all understanding takes place. In fact, he went further, stating that everything that can be understood is based in language: ‘all understanding is interpretation, and all interpretation takes place in the medium of a language’ ([11], p. 389). Even when we feel we are rendered speechless and use the familiar phrase ‘I haven’t got the words…’, Gadamer reminds us that we often don’t end there, but rather *begin* to go on and describe what we wish to express ([10], p. 72). We may doubt the ability of words to express what we wish to say, but we return to them to try to express ourselves. It can be no surprise then, that the term ‘dialogue’ features throughout Gadamer’s philosophy.

The essence of dialogue for Gadamer is in its etymology (dia (through) and logos (reason/ meaning). Dialogue for Gadamer is always *a shared search for truth*. Only in dialogue can truth unfold. He applies the concept to the doctor-patient relationship, explaining what the essence of true dialogue is:

In the somewhat tense relationship between doctor and patient, dialogue can help a great deal. But this dialogue is only really successful when it takes place almost as if it were a normal conversation. In our everyday lives we fall into discussions which are sustained by everyone involved rather than led by one person in particular. And this is how it should be even for the special form of dialogue that takes place between doctor and patient ([9], p. 137).

While listening is certainly an essential part of the doctor-patient relationship and an important skill for the physician to master, Gadamer’s concept of dialogue doesn’t single out listening, but rather focuses on the equal back and forth of conversation. He uses the analogy of two wood cutters working together with a large saw, each pulling and pushing in rhythm and with the correct pressure on the saw to cut the wood. The image demonstrates just how sensitive dialogue is— a coordinated movement, where there is give and take, a careful back and forth between two parties working in rhythm to achieve a result.

Gadamer’s rehabilitation of the concept of prejudice (Vorurteil) reminds us that understanding is always shaped by prior assumptions and expectations. In clinical encounters, awareness of this interpretive situatedness may itself be beneficial for the physician. Gadamer describes understanding as a form of “play” (Spiel), in which the interpreter participates in a movement of meaning that is not entirely under conscious control. Recognising this dimension of play may allow clinicians to experience dialogue not solely as a task requiring mastery, but as an encounter that invites curiosity, openness, and reflection on one’s own standpoint. Such awareness does not eliminate pre-understanding, which Gadamer argues is impossible, but it may enable a more flexible relationship to one’s own assumptions. Here, pre-understanding refers to the background assumptions, prior experiences, and interpretive expectations that shape how clinical situations are perceived and understood.

For Gadamer, interpretation is always situated and therefore cannot claim absolute objectivity. But this does not mean that any one interpretation is as good as the next. Understanding develops through participation in dialogue— in which one’s own perspective is placed at risk through encounter with the other. In this process, interpretations are not simply asserted but are tested in relation to what proves meaningful, coherent, and illuminating within the shared situation.

In clinical practice, such ‘testing’ occurs through the unfolding of conversation, the patient’s response, and the physician’s reflective judgement regarding what genuinely clarifies the situation. Hermeneutic understanding therefore remains disciplined not by methodological detachment but *by attentiveness to what the dialogue itself discloses.* For the physician, this may transform interpretation from a demand for certainty into a form of engaged practical judgement, allowing professional responsibility to coexist with interpretive humility. In this sense, hermeneutic attentiveness may contribute to professional self-care by offering clinicians a way of situating themselves within the clinical encounter that allows both engagement and reflective distance. In the world of medicine this ideal has important practical parallels: engaging in dialogue with the patient ensures that the patient understands the situation, and avoids the growing problem of health literacy in patients [21].

Such an exchange also avoids the possibility that the patient might feel unheard. Medical research shows us that there is a distinction between ‘being heard’ and the *impression* that one is being heard. In a Johns Hopkins Bloomberg School of Public Health interview, Dr. Mary Catherine Beach, who studies patient-provider communications reports that ‘When patients don’t feel heard by their doctors, there’s an erosion of trust that can lead to serious health consequences—even if clinicians have their patients’ best interests in mind’ [6].

Thus it would seem then that clinical listening operates on two levels: the physician must understand the patient’s concerns, and the patient must *experience themselves as being listened to*,* and understood*. Ideally of course, both coincide. A Gadamerian approach to dialogue helps bring these together, because it treats understanding as a shared, relational process rather than a one-sided transmission of information. Unsurprisingly, there is a direct correlation between patient satisfaction and the time spent by the doctor engaging in the kind of dialogue that Gadamer describes. A 2022 article found that ‘the factors that affect the communication satisfaction level are the duration of communication, time allowed for expressing questions and interest in patients’ personal situation’ [17].

What element (or elements) constitutes ‘interest in patients’ personal situation’? By encouraging the physician to move beyond the mere collection of symptoms, case histories, or clinical data, a mutual space of interest is created. I advocate that adopting this mindset can lessen the feeling of isolation and the pressure of ‘always having to be in control’ that can lead to burnout. In everyday cases — a cold or a rash— adopting this mindset may seem unnecessary. But Gadamer’s point is that *even there*, if the physician truly engages, allowing for the patient’s meaning to take shape in the space between them, the encounter becomes more than mechanical. It restores the human and relational heart of medicine.

A potential criticism of Gadamer’s dialogical model, articulated by Habermas, concerns the influence of *Herrschaft* (relations of power) on communication [7], while Michel Foucault has demonstrated how medical knowledge itself is historically embedded within systems of authority [8]. The clinical encounter is necessarily shaped by asymmetries of knowledge, authority, and patient vulnerability, conditions that may appear to limit the possibility of fully open dialogue. Gadamer does not deny such asymmetries, but suggests that authority, when grounded in recognised expertise, need not preclude understanding. Rather, awareness of imbalance may heighten the physician’s attentiveness to creating conditions in which the patient feels able to speak. When dialogue enables the patient to articulate their experience with sufficient clarity, the physician may recognise that understanding has emerged despite structural constraints. Such recognition may itself contribute to professional self-care, as the clinician can judge that the encounter has fulfilled an important dimension of good practice: the creation of a communicative space in which the patient’s perspective can meaningfully inform clinical understanding.

Dialogue leads to what Gadamer calls a *Horizontverschmeltzung*, which has been translated into English as a ‘fusion of horizons’. It is another of the key concepts of Gadamer’s philosophical hermeneutics. Suspending pre-understandings (training, diagnostic expectations, prejudices) does not imply detachment, but a deepened attentiveness to the patient’s lived experience. Encouraging the patient to express how their illness *makes them feel*, or what effect it has on their life helps the physician to arrive at a shared understanding—a melting of two horizons. Again, this philosophical approach has clear parallels within modern medical research:

HCPs who demonstrate genuine concern for the physical and emotional well-being of their patients tend to provide more effective, patient- focused care that considers the individual’s needs, resulting in better results. Additionally, compassion can help foster trust between patients and HCPs, which in turn leads to more adherence to treatment plans and safety recommendations [1].

If done consciously and mindfully, I argue that this act not only benefits the patient, but reciprocally can be harnessed by the physician to renew the sense of connection and purpose that first drew them to medicine, countering the erosion of meaning that fuels burnout.

### Reflective practice

- Reflect on a clinical encounter from the patient’s horizon. What might you have missed?
- Personal ‘fusion’ practice: Identify areas where your professional and personal identities can be reconciled rather than compartmentalized.
- Dialogical journaling: Write dialogues between your ‘clinical self’ and your ‘human self’ after difficult cases. Keep them and re-read these regularly. Each time you return to these, jot down how your perspective has evolved.
- Practise genuinely listening to patients and peers without agenda — cultivating presence as self-renewal.

### From dialogue to sensus communis—from sensus communis to phronesis

We have seen that dialogue holds a central place in Gadamer’s philosophy, because it emerges from language—which is the very foundation of his hermeneutical vision and the source from which all understanding and meaning flow. In *Truth and Method*, Gadamer painstakingly traces the history of the term *sensus communis*. While he sometimes translates *sensus communis* as ‘common sense’, he is, at the same time, careful to explain that it is much more than this. For Gadamer, *sensus communis* isn’t a gift that someone is born with, but rather a mindset that is developed over time, through experience with things outside ourselves. While dialogue focuses on the relationship between individuals, *sensus communis* is communal. *Sensus communis* then, is a *cultivated ability*—a shared judgment, taste, and common sense that evolves as a relational capacity to one’s environment or culture. It is the evolution of one’s judgment through interaction with one’s social and cultural context:

There is something immediately evident about grounding philological and historical studies and the ways the human sciences work on this concept of the sensus communis. For their object, the moral and historical existence of humanity, as it takes shape in our words and deeds, is itself decisively determined by the sensus communis ([11], p. 22).

*Sensus communis* is the capacity to imagine oneself within the moral and social world of others. There is no science or methodology to be learned—‘it’ (*sensus communis*) happens only where there is an openness to participation, with the understanding that this participation means letting go of self-certainty (or even self-righteousness) in favour of a more universal, humanistic understanding. Again, the concept has concrete applications to the doctor-patient relationship: ‘the physician must know not only the nature of the soul but the nature of the ‘whole’ to be able really to treat the patient’s experience of lack, of illness and pain’ ([9], p. 88).

Because it is an acquired skill over time, the concept of *sensus communis* can feel discouraging for the trainee doctor, as it highlights a gap between where the trainee currently stands and the wisdom of older, seasoned clinicians. However, on deeper reflection, *sensus communis* can be seen not as a barrier but as a goal — a quality developed through attentive practice, reflection, and genuine engagement with patients and colleagues. It reminds the trainee that *medicine is not only a technical science but also a profoundly human* art, and that becoming a good doctor involves developing one’s judgment in alignment with shared standards and norms, seeing beyond purely personal or technical perspectives. In the following article, medical research shows how Gadamer’s idea of *sensus communis* plays out in practice, identifying four elements that constitute the ideal doctor-patient relationship —mutual knowledge, trust, loyalty, and regard. These elements exemplify *sensus communis* because they represent culturally accepted, shared expectations of the doctor-patient relationship—a quasi-mutual understanding that is recognised and valued, yet nowhere written down or codified in formal guidelines:

The doctor-patient relationship involves vulnerability and trust. It is one of the most moving and meaningful experiences shared by human beings. However, this relationship and the encounters that flow from it are not always perfect […] This unique relationship encompasses 4 key elements: mutual knowledge, trust, loyalty, and regard. Knowledge refers to the doctor’s knowledge of the patient as well as the patient’s knowledge of the doctor. Trust involves the patient’s faith in the doctor’s competence and caring, as well as the doctor’s trust in the patient and his or her beliefs and report of symptoms. Loyalty refers to the patient’s willingness to forgive a doctor for any inconvenience or mistake and the doctor’s commitment not to abandon a patient. Regard implies that the patients feel as though the doctor likes them as individuals and is “on their side.” These 4 elements constitute the foundation of the doctor-patient relationship [4].

For the seasoned doctor who feels stretched and burnt out, the concept of *sensus communis* might seem hollow, even a reprimand that they have not engaged fully with their patients. Yet, I argue here that this shared human sense may in fact offer a path back to meaning. Burnout often arises when a doctor’s sense of connection — to patients, colleagues, and their own purpose — becomes eroded. It is not surprising that ‘social isolation is associated with increased burnout, suicidal ideation, and lower professional fulfilment, and is more common among US physicians than workers in other fields’ [32]. *Sensus communis* reminds us that good medicine depends not just on individual resilience, but on *shared* understanding, empathy, and recognition of our common humanity. For the burnt-out doctor, revisiting this idea may help rekindle a sense of belonging and moral purpose within the practice of medicine itself. *Sensus communis* recognises that doctors are human too and experience suffering. *Sensus communis* is the realisation that burnout is not a failure of moral sense, but a signal that the system and individual have been stretched beyond what this shared humanity can sustain. By engaging again with colleagues, patients, and the wider community, the burnt-out doctor might begin to recover that sense of purpose and belonging.

Following on from Gadamer’s concept of *sensus communis* is his adoption of the Aristotelian idea of *phronesis—*a kind of ‘practical wisdom’ that is directed towards a certain problem or event. This ‘practical wisdom’ is outside the yardstick of rational knowledge or facts, and is more than a sense of judgement. Gadamer writes of *phronesis*:

Rather there is a positive ethical motif involved that merges into the Roman Stoic doctrine of the sensus communis. The grasp and moral control of the concrete situation require subsuming what is given under the universal—that is, the goal that one is pursuing so that the right thing may result ([11], p. 21).

*Sensus communis* is the shared, cultivated sense of judgment that makes *phronesis* possible. *Phronesis* is a kind of ‘practical wisdom’ and can be understood as the ability to discern and enact the right response at the right time. For the clinician, Gadamer describes *phronesis* as ‘an awareness appropriate to a particular situation, like that in which diagnosis, treatment, dialogue and the participation of the patient all come together’ [9]. In Gadamer’s thought, this is the ethical centre of understanding: *the wisdom to apply what we know to the uniqueness of each human being.* As any experienced doctor will tell you, what is best for the body may not always be best for the person. For Gadamer, *phronesis* represents yet again that kind of truth that cannot be reduced to method because it is instinctive, dealing with particularity, demanding a sense of tact, and a strong sense of other:

What takes place here between doctor and patient is a form of attentiveness, namely the ability to sense the demands of an individual person at a particular moment and to respond to those demands in an appropriate manner. It is in these terms that we must understand what is involved in therapeutic dialogue ([9], p. 138).

In medicine, this distinction is vital. Science and evidence-based medicine provide one kind of knowledge — measurable, generalisable, protocol-driven. Adopting a *phronesis* mindset means that the doctor acknowledges and embraces the fact that patients rarely present as neatly bounded ‘cases’ that fit perfectly into predetermined structures. They arrive with their fears, histories, families, values, and vulnerabilities. In his book *Whole Person Care* Dr. Tom Hutchinson describes bringing two sherry glasses, a bottle of sherry and two cigars to share with his wife in hospital after she had been diagnosed with sarcoidosis: ‘We certainly felt better but is this the recommended treatment for sarcoidosis or any other disease? Well of course not, but was it the right treatment for the people we were then?’ [14]. His implicit answer is yes. The example is, of course, deliberately vivid, yet it makes the point with striking clarity—while diagnostic tools can tell the physician what is happening with the soma, *phronesis can determine what should be done.*

### Gadamer in practice—Balint groups

Perhaps medicine’s most concrete realisations of Gadamerian hermeneutics in practice are to be found in Balint groups, first developed in the 1950s by psychoanalyst Michael Balint. Created to afford doctors a space to meet with their peers and reflect on their relationships with patients, these groups embody the very process Gadamer describes: understanding through dialogue, interpretation, and openness to the perspective of the other. In his seminal work *The Doctor*,* His Patient and the Illness* (1957) Balint embodies the Gadamerian concept of *sensus communis* and *phronesis* in two sentences:

The most frequently used drug in general practice is the doctor himself. It is not only the bottle of medicine or the box of pills that matters; the way the doctor gives them, the words he uses, the atmosphere he creates—all play a part in their effect [2].

In the Balint model, meaning arises not from detached analysis but from shared reflection — a fusion of horizons between clinician, patient, and peers. This collective inquiry transforms the clinical encounter from a technical act into an interpretive one, cultivating precisely the kind of *sensus communis* and *phronesis* that Gadamer holds essential for genuine understanding and ethical action. In this way, Balint groups stand as a living, medical form of hermeneutic practice — a structured space where physicians learn, through conversation, to interpret not only their patients’ experiences, but also their own.

### Reflective practice

- Mindful decision journaling: After difficult choices, reflect on what guided you, what felt right, and what you learned.
- After clinical encounters, take a few minutes to write not about *what* you did, but *what it felt like* — for you and for the patient. Ask: ‘*What might this moment have felt like from their side? What did it feel like for me to be in that role?’* Over time, this kind of reflective writing restores the *mutuality* of clinical encounters — reminding the doctor that they are not merely a provider, but part of a shared human moment.
- Perspective-taking conversations: Join (or start) a reflective group where clinicians discuss the emotional and moral dimensions of patient care. Such spaces enact *sensus communis* in real time: doctors practice understanding one another’s moral perspectives and reaffirm shared values, reducing the isolation that fuels burnout.

## Conclusion and limitations

In this article I argue that Gadamer’s philosophical hermeneutics have real world value for physicians. Adapting his philosophical and deeply phenomenological outlook invites a reshaping of the work environment, leading to skills that inevitably encapsulate what it means to be a ‘good doctor’, or at the very least, to what patients believe are the most important aspects or traits that a doctor should possess.

Gadamer himself understood that his hermeneutics have a positive role to play in healthcare. His book *The Enigma of Health*, a collection of essays and lectures taken over decades show just how engaged Gadamer was with the practice of medicine. I argue here that Gadamer’s outlook, if adopted, may help to reframe the clinical environment in such a way as to sustain and help the physician in retaining a sense of self-leadership—leading to an enhanced sense of wellbeing.

Gadamer’s philosophy offers a counterweight to burnout and moral injury by reminding clinicians that medicine is not only technical work but human dialogue.

Medical science and the advancements it has made are to be celebrated. However, the advancements that have been made often lead to a focus on medicine as a primarily diagnostic endeavour, as something to be ‘solved’. The art to medicine is acknowledging that there are two equally strong components that facilitate health, and that that second element—the dialogue and engagement with the patient, potentially opens up a world of knowledge for the doctor—a *sensus communis* that is shaped by the assimilation of many different horizons, leading to a clearer picture of the whole—of humanity itself. Here I have argued that such an awareness frees the physician from the impossible demand for certainty.

It must also be acknowledged that many of the pressures contributing to physician burnout arise from structural conditions within contemporary healthcare systems: limited consultation times, increasing administrative burdens, and institutional priorities that can reduce clinical encounters to procedural efficiency. These systemic challenges cannot be solved by individual physicians alone. The reflections offered in this article are therefore not presented as a substitute for structural reform, but rather as a complementary perspective. While systemic change remains essential, cultivating interpretive attentiveness and dialogical engagement within the clinical encounter may help physicians sustain a sense of meaning and professional identity even within constrained environments.

There is no doubt that A.I. will redefine medicine and medical practice in the coming years. This technological advance holds enormous potential, but it also demands vigilance, for it threatens to deepen an already dangerous imbalance—the privileging of what can be measured over what can be understood. Once again, medicine risks being seduced by the technical, mistaking data for discernment and efficiency for care. It may be argued that the very ‘advancements’ that have been made in medicine have contributed to the epidemic of burnout and moral injury among physicians. Now more than ever, a Gadamerian mindset is needed, understanding that illness and healing, are, in first place, a deeply human endeavour.

## Data Availability

Not applicable; no datasets were generated or analysed in this study.

## Declarations

This article constitutes an original philosophical and theoretical contribution authored solely by the author and did not involve human participants or third-party research.
